# FGF16 regulated by miR-520b enhances the cell proliferation of lung cancer

**DOI:** 10.1515/med-2021-0232

**Published:** 2021-03-15

**Authors:** Wenfeng He, Xia Liu, Zhijie Luo, Longmei Li, Xisheng Fang

**Affiliations:** Department of Oncology, The Second Affiliated Hospital of Guangzhou University of Chinese Medicine, Guangdong Provincial Hospital of Chinese Medicine, Guangzhou, Guangdong, 510145, China; Department of Medical Oncology, Guangzhou First People’s Hospital, School of Medicine, South China University of Technology, Guangzhou, Guangdong, 510180, China; Department of Oncology, The First Affiliated Hospital of Guangzhou University of Chinese Medicine, Guangzhou, Guangdong, 510405, China

**Keywords:** FGF16, miR-520b, growth, lung cancer

## Abstract

FGF16 is implicated in the progression of some specific types of cancers, such as embryonic carcinoma, ovarian cancer, and liver cancer. Yet, the function of FGF16 in the development of lung cancer remains largely unexplored. In this study, we present the novel function of FGF16 and the regulation of miR-520b on FGF16 in lung cancer progression. In clinical lung cancer tissues, FGF16 is overexpressed and its high level is negatively associated with the low level of miR-520b. Furthermore, both the transcription and translation levels of FGF16 are restrained by miR-520b in lung cancer cells. For the regulatory mechanism investigation, miR-520b is able to directly bind to the 3′-untranslated region (3′UTR) of FGF16 mRNA, leading to its mRNA cleavage in the cells. Functionally, miR-520b reduces the growth of lung cancer and its inhibitor anti-miR520b is able to promote the growth through competing endogenous miR-520b. Moreover, FGF16 silence using RNA interference is capable of doing great damage to anti-miR-520b-accelerated growth of lung cancer. Thus, our finding indicates that FGF16 is a new target gene of miR-520b in lung cancer. For lung cancer, FGF16 may serve as a novel biomarker and miR-520b/FGF16 may be useful in clinical treatment.

## 1 Introduction

As a big family of polypeptide growth factors, fibroblast growth factors (FGFs) contain structurally related 22 members and share high amino acid identity in vertebrates. FGFs can affect cell proliferation, migration, and differentiation in embryonic development. Although FGFs play important roles in the response to injury and tissue repair in the adult organism, some ectopically expressed FGFs can affect the tumorigenesis [1]. FGF16 is the sixteenth member of FGF family [2]. Four FGFs such as FGF16, FGF10, FGF17, and FGF18 can increase the survival of human embryonic carcinoma cells [3]. FGF16 together with WNT signaling enhance the development of ovarian cancer [4]. Some FGFs, such as FGF1, FGF3, FGF4, and FGF16, are unstable and their stabilities can function in the regulation of FGF signaling [5]. During combating obesity, FGF16 is identified as a novel inducer activating brown adipose tissue (BAT) and inducible BAT [6]. FGF16 can be regulated by miR-520f in liver cancer development [7]. However, the function of FGF16 and its regulation in lung cancer progression are still unclear.

At the post-transcriptional level, microRNAs (miRNAs), members of non-coding RNA family, can promote mRNA degradation or translation repression of their target genes [8,9]. The levels of lots of miRNAs are altered during the development of cancers [10]. miRNAs are involved in many processes, such as proliferation, differentiation, or carcinogenesis [11,12,13]. For miRNAs, both oncogenic function and tumor suppressor role in cancer development are revealed [14,15,16,17,18]. IFN-γ can induce miR-520b to reduce an NKG2D ligand expression [19]. miR-520b-targeted HBXIP and IL-8 can affect breast cancer progression [20]. miR-520b takes part in the liver cancer induced by HBx and survivin [21]. HBXIP/TFIID/Lin28B/miR-520b/HBXIP feedback loop is involved in the promotion of breast cancer [22]. In breast cancer, CD46 is one of the target genes of miR-520b/e [23]. miR-520b can increase the chemotherapy sensitization of liver cancer cells [24]. miR-520b-mediated MBD2 functions in the progression of glioma [25]. miR-520b can also reduce CD44 expression during head-neck cancer development [26]. miR-520b-associated TET1 is reported to play a key role in hepatocarcinogenesis [27]. Cyclin D1 is one target gene of miR-520b in glioblastoma [28]. miR-520b is able to prohibit lung cancer via regulating HDAC4 [29]. miR-520b targets Capn4, further modulating Wnt/β-catenin pathway to affect prostate cancer [30]. GATA6/miR-520b/CREB1 is involved in gastric cancer [31]. MLK3, as a novel target gene of miR-520b, functions in liver cancer [32]. In non-small cell lung cancer miR-520b can repress the growth and migration via regulating CHAF1A [33]. Yet, the role of miR-520b and its novel target gene in lung cancer need more investigation.

In this study, we present the novel function of FGF16 in lung cancer and its regulation factor at the post-transcription level. We find that FGF16 is a novel target gene of tumor suppressor miR-520b in lung cancer. Notably, FGF16 is highly expressed in lung cancer and it is able to reverse miR-520b-inhibited lung cancer. Our study provides a promising evidence for FGF16 as the potential target in anti-lung cancer drug study.

## 2 Materials and methods

### 2.1 Patient tissues

The Guangzhou First People’s Hospital (Guangzhou, China) is responsible for the collection of 30 paired lung cancer tissues and their noncancerous tissues derived from 30 lung cancer patients. The patient information is listed in Table S1. The use of clinical lung cancer tissues in this study has gained the approval from patients. Research Ethics Board of the Guangzhou First People s Hospital (Guangzhou, China) has approved the study protocol.

### 2.2 RNA extraction, qRT-PCR, and RT-PCR

TRIzol Reagent (Invitrogen, USA) was applied for the RNA extraction from human clinical lung cancer tissues, their noncancerous tissues, or lung cancer A549 and H1299 cells. Reverse transcription was performed through TransScript^®^ First-Strand cDNA Synthesis SuperMix (TransGen Biotech, China) followed by manufacturer’s instructions. For miR-520b analysis, poly(A) polymerase (Ambion, USA) was used to polyadenylate total RNA and was used for the template. TransStart^®^ Top Green qPCR SuperMix (TransGen Biotech, China) was applied for qRT-PCR assay of miR-520b and FGF16 in clinical lung cancer tissues and their noncancerous tissues. GAPDH was used to normalize FGF16. U6 was used to normalize miR-520b. The primers to amplify FGF16 or GAPDH are listed in Table S2.

### 2.3 Cell line

Lung cancer cell lines A549 and H1299 were obtained from the American Type Culture Collection (ATCC, USA). Both these two cell lines were cultivated in Dulbecco’s modified Eagle’s medium with 10% fetal bovine serum (FBS; Gibco, USA) and maintained at 37°C with 5% CO_2_.

### 2.4 Immunoblotting analysis

A549 and H1299 cells were seeded into 6-well plates and maintained for 24 h. Different doses of miR-520b were transfected into cells using lipofectamine 2000 reagent (Invitrogen, USA). Post 48 h, the cultured cells were harvested for FGF16 analysis. After the cells were washed by ice-cold PBS, RIPA buffer was applied to lyse the cells and isolate total protein. After the electrophoresis of SDS–PAGE gel was performed, PVDF membranes (Millipore, USA) were blocked with 5% skim milk solution of PBS. In this study, the primary antibodies included anti-FGF16 (Abcam, USA) or anti-β-actin (Abcam, USA). After the incubation of horseradish peroxidase (HRP)-conjugated secondary antibodies for 2 h, the membrane was visualized by ECL (Millipore, USA).

### 2.5 Luciferase reporter gene assays

The lung cancer A549 cells were cultured on 24-well plates overnight. Lipofectamine 2000 (Invitrogen, USA) was used to transfect miR-520b and/or luciferase vectors into the cells. After 48 h, the luciferase activities were assessed through Dual-Luciferase Reporter Assay System (Promega, USA) following manufacturer’s instructions. Renilla luciferase activity was used to normalize firefly luciferase activities of pGL3-FGF16-wt and pGL3-FGF16-mut. All experiments were repeated thrice at least.

### 2.6 Proliferation analysis

A549 cells (3,000 cells/well) were grown on 96-well plates for 24 h. miR-520b, anti-miR-520b, or anti-miR-520b and si-FGF16 (RiboBio, Guangzhou, China) was transfected into the cells with at least three replicates through lipofectamine 2000. At the indicated time point (0, 24, 48, or 72 h), 10 μL MTT (5 mg/mL) was supplied into the wells. The absorbance values at OD_490nm_ were determined by the absorbance reader.

### 2.7 Statistical analysis

Each experiment was performed in triplicate at least. All data were analyzed by comparing mean values (±SD) using Student’s *t*-test. The significance of different groups’ differences was assumed as not significant (NS); *P < 0.05; **P < 0.01; ***P < 0.001. The relationship of the expression level of FGF16 and miR-520b in 30 cases of human lung cancer tissues was assessed through Pearson’s correlation coefficient.

## 3 Results

### 3.1 FGF16 is increased and negatively correlated with tumor suppressor miR-520b in clinical lung cancer tissues

**Figure 1:**
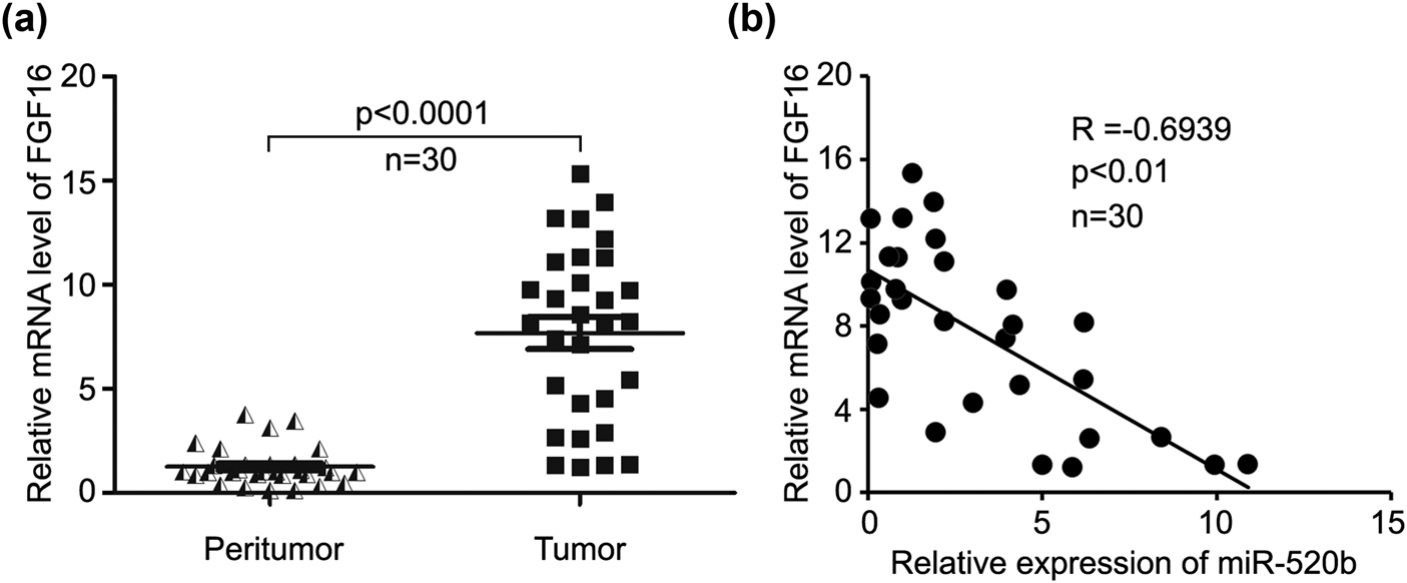
FGF16 is increased and negatively correlated with tumor suppressor miR-520b in clinical lung cancer tissues. (a) qRT-PCR assay was used to analyze the expression of FGF16 in 30 cases of clinical lung tissues and their noncancerous tissues. (b) Pearson’s correlation coefficient was applied for the analysis of the relationship of FGF16 with miR-520b in 30 cases of human lung cancer samples (*R* = −0.6939).

As the sixteenth member of FGF family, FGF16 is involved in the progression of some specific types of cancers, such as embryonic carcinoma, ovarian cancer, and liver cancer [3,4,7]. However, its expression and its role in lung cancer development are largely unknown. First, we tested the level of FGF16 in 30 paired lung cancer tissues and noncancerous tissues. Our data showed that FGF16 is markedly overexpressed in clinical lung cancer samples (Figure 1a). miRNAs can induce mRNA degradation or translation repression of their target genes, regulating the expressions of multiple genes at the post-transcriptional level [8,9]. miR-520b can serve as a tumor suppressor in some kinds of cancers such as breast cancer, liver cancer, glioma, head-neck cancer, colorectal cancer, spinal osteosarcoma, gastric cancer, glioblastoma, lung cancer, and prostate cancer [20,21,25,26,30,34,35,36]. We predicted the target genes of miR-520b through online informatics sites, such as TargetScan (http://www.targetscan.org/), and then found that FGF16 was one of the target genes of miR-520b. To clarify the relationship of FGF16 and miR-520b in lung cancer, we further analyzed the level of miR-520b using above 30 cases of human lung cancer tissues. Our data revealed that high expression of FGF16 is closely associated with low level of miR-520b in these 30 cases of human lung cancer samples (*R* = −0.6939, *P* < 0.01, Figure 1b). In conclusion, FGF16 is negatively correlated with miR-520b in human clinical lung cancer samples.

### 3.2 Within lung cancer cells the expression of FGF16 is restrained by miR-520b

**Figure 2:**
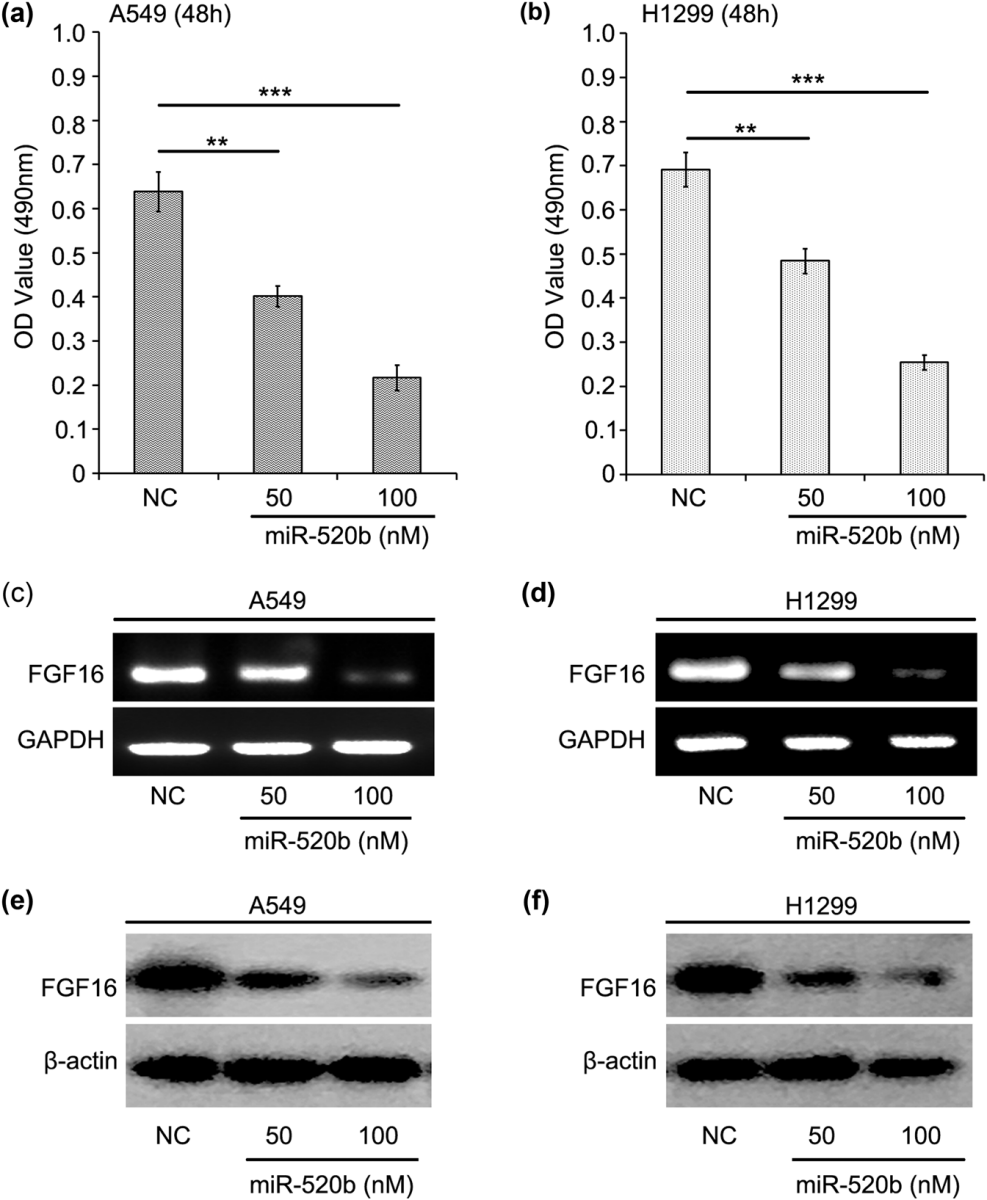
Within lung cancer cells the expression of FGF16 is restrained by miR-520b. (a and b) The effects of different doses of miR-520b (50 and 100 nM) on cell proliferation in both A549 cells and H1299 cells were analyzed using MTT assay. (c and d) RT-PCR assay was used to test the change in mRNA level of FGF16 after lung cancer A549 and H1299 cells were transfected with increasing concentration of miR-520b (50 and 100 nM). (e and f) Immunoblotting assay was performed to evaluate the alteration of protein level of intrinsic FGF16 in miR-520b (50 and 100 nM) treated A549 and H1299 cells.

First, we evaluated the effect of different doses of miR-520b on cell proliferation in both A549 cells and H1299 cells using MTT assay. We found that increasing dose of miR-520b could obviously inhibit the cell proliferation (Figure 2a and b). Next, we tried to investigate the modulation of miR-520b on FGF16 in lung cancer A549 and H1299 cell lines through RT-PCR and immunoblotting assays. At the first time, we revealed that the transcription of FGF16 could be markedly reduced by miR-520b at the high dose treatment in A549 cells (Figure 2c). In further analysis, our data showed that the protein level of FGF16 was also decreased in miR-520b-transfected A549 cells (Figure 2e). To further confirm the results obtained from lung cancer A549 cell line, we treated another lung cancer cell line H1299 with miR-520b and analyzed the change in RNA and protein levels of FGF16. As expected, miR-520b also caused a decrease in RNA and protein levels of FGF16 in H1299 cell line (Figure 2d and f). All our results indicate that the FGF expression is retrained by tumor suppressor miR-520b in lung cancer cells.

### 3.3 FGF16 is identified as a novel target gene of miR-520b in lung cancer

**Figure 3:**
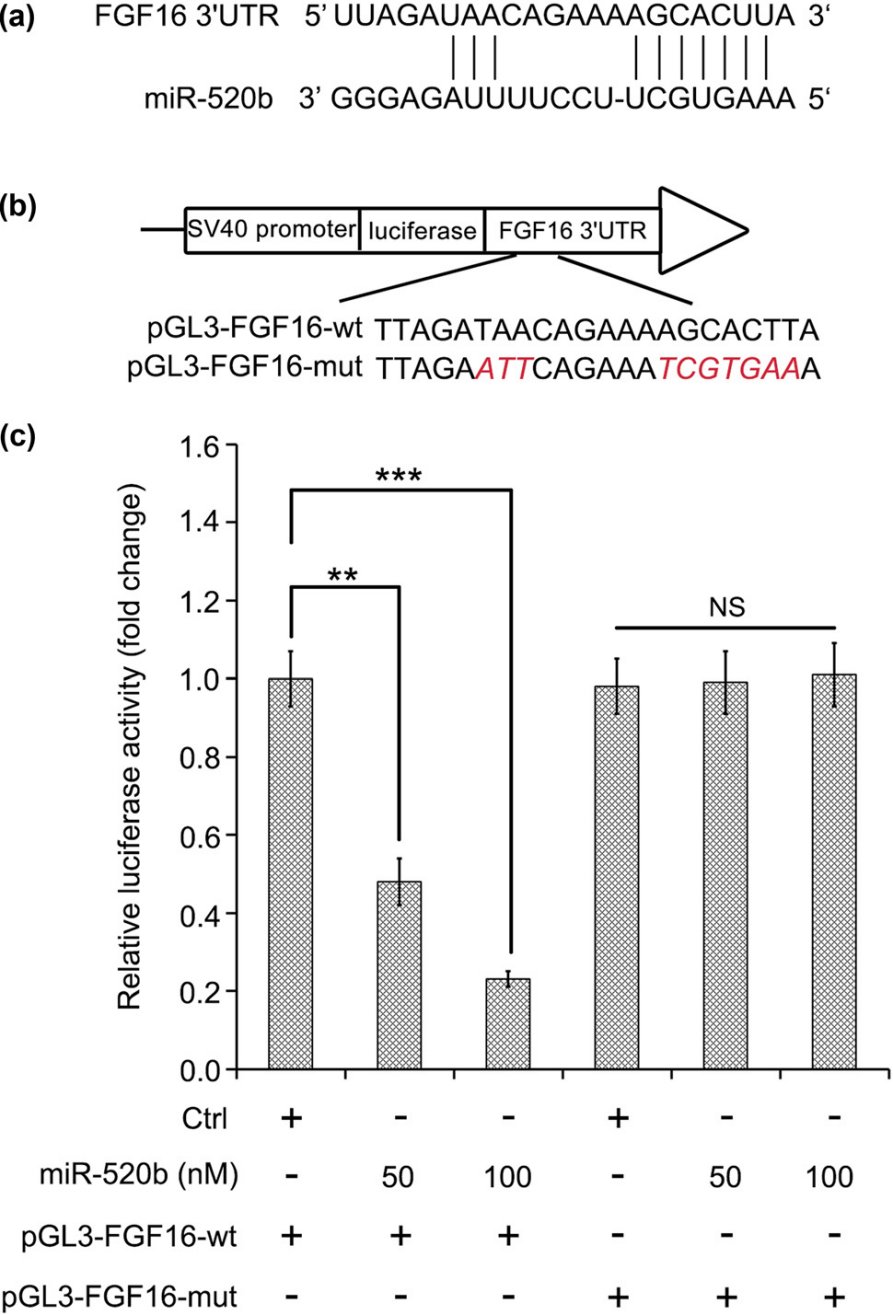
FGF16 is identified as a novel target gene of miR-520b in lung cancer. (a and b) The binding model of the seed region of FGF16 mRNA 3′UTR to miR-520b is presented. The binding site of FGF16 mRNA 3′UTR (wild type or mutant) with miR-520b is cloned into luciferase reporter vector, pGL3-control. (c) The luciferase activities of pGL3-FGF16-wt or pGL3-FGF16-mut were examined when lung cancer A549 cells were treated with elevated dose of miR-520b. Student’s *t*-test: NS, non-significant; **P < 0.01; ***P < 0.001.

According to the prediction information from TargetScan (http://www.targetscan.org/), we cloned the wild and mutant 3′ untranslation region (3′UTR) fragment of FGF16 containing seeding site with miR-520b into pGL3-control vector (namely pGL3-FGF16-wt and pGL3-FGF16-mut; Figure 3a and b). Then, we investigated the mechanism by which miR-520b decreased the mRNA level of FGF16 in lung cancer cells. We found that along with the increase in miR-520b concentration, the decrease in luciferase activities of pGL3-FGF16-wt was more obvious. However, miR-520b had no effect on the luciferase activities of pGL3-FGF16-mut in lung cancer A549 cells (Figure 3c). Therefore, our data imply that FGF16 is a direct target gene of miR-520b in lung cancer.

### 3.4 FGF16 can hold back miR-520b-suppressed growth of lung cancer

**Figure 4:**
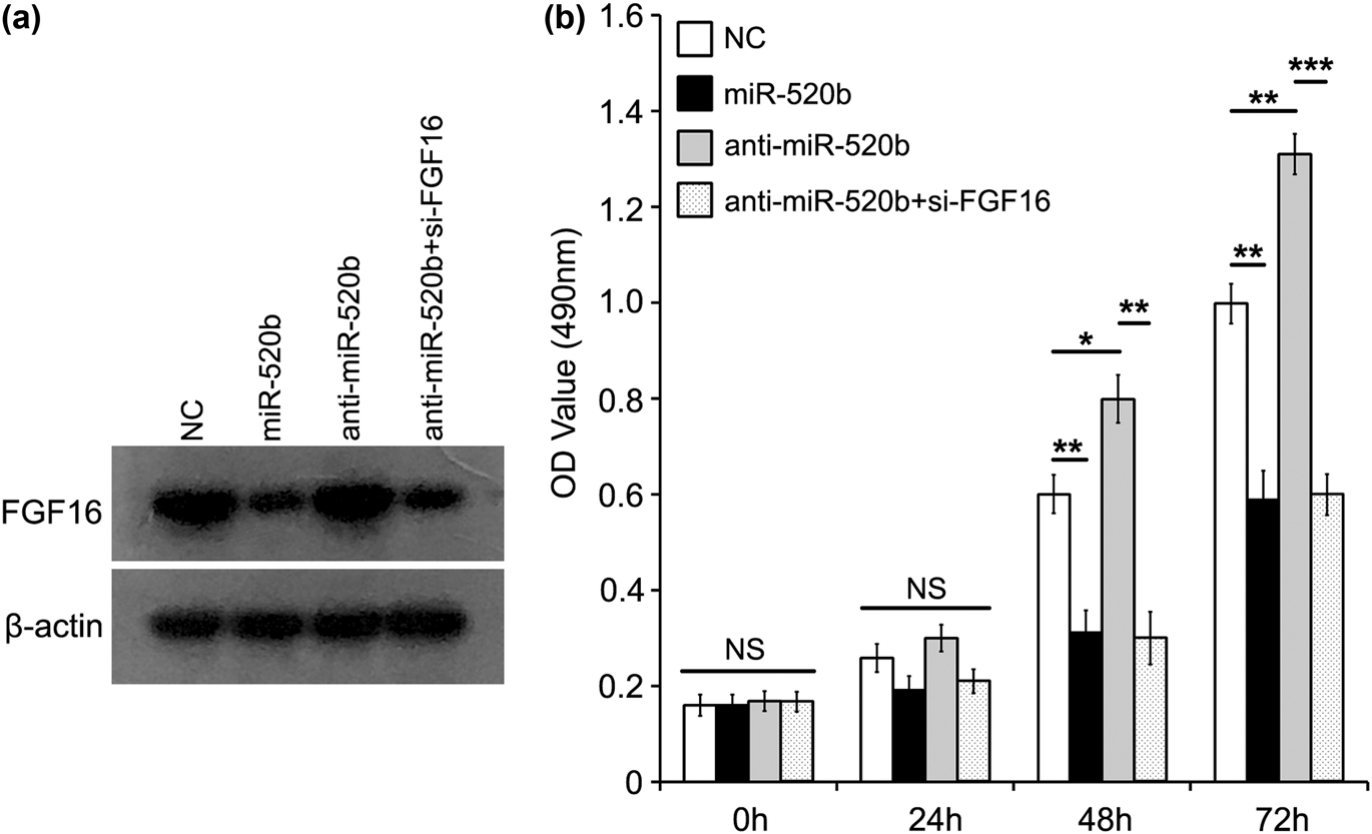
FGF16 can hold back miR-520b-suppressed growth of lung cancer. (a) Immunoblotting assay was performed to analyze the level of intrinsic FGF16 after A549 cells were treated with miR-520b (100 nM), anti-miR-520b (100 nM), and anti-miR-520b (100 nM)/si-FGF16. (b) In the above different groups, MTT analysis was used to test A549 cell proliferation. Student’s *t*-test: NS, non-significant; *P < 0.05; **P < 0.01; ***P < 0.001.

For the function investigation, we are interested in the role of FGF16 in miR-520b-regulated growth of lung cancer. We first evaluated the level of FGF16 in lung cancer A549 cells after the cells were treated with miR-520b, anti-miR-520b, or anti-miR-520b/si-FGF16 via immunoblotting analysis (Figure 4a). Through 3-(4,5-dimethyl-2-thiazolyl)-2,5-diphenyl-2-*H*-tetrazolium bromide (MTT) assay, we observed the obvious inhibition of lung cancer cell proliferation induced by miR-520b. However, the inhibitor of miR-520b (anti-miR-520b) can markedly promote the growth. Taken a step further, knockdown of FGF16 using si-FGF16 could destroy the anti-miR-520b-induced increase in cell proliferation (Figure 4b). Taken together, our results suggest that miR-520b-regulated FGF16 is involved in the growth of lung cancer.

## 4 Discussion

As one of the 22 members of FGF family in vertebrates, FGF16 can promote the survival of human embryonic carcinoma cells [3]. FGF16 in combination with WNT pathway is able to drive the ovarian cancer progression [4]. miR-520f-mediated FGF16 is revealed to involve hepatocarcinogenesis [7]. Yet, the function of FGF16 in the development of lung cancer is not determined. Dozens of studies identify miR-520b as a tumor suppressor in some specific types of cancers. miR-520b-targeted HBXIP/IL-8 can affect breast cancer progression [20]. miR-520b takes part in HBx/survivin-induced liver cancer [21]. miR-520b-mediated MBD2 functions in the progression of glioma [25]. miR-520b is able to reduce CD44 expression during head-neck cancer development [26]. Cyclin D1 is one target gene of miR-520b in glioblastoma [28]. miR-520b can prohibit lung cancer via regulating HDAC4 [29]. miR-520b targets Capn4, further modulating Wnt/β-catenin pathway to affect prostate cancer [30]. In addition, miR-520b is a mediator of other noncoding RNA affecting cancer development. miR-520b/EZH2 mediates circular RNA TTBK2-accelerated glioma [37]. miR-520b/USP21 is involved in lncRNA FGD5-AS1-enhanced oral squamous cell carcinoma [38]. As an identified tumor suppressor, miR-520b derived from exosomes of normal pancreatic fibroblasts has the potential therapeutic effect on pancreatic cancer [39]. However, the role of miR-520b and its novel target genesin lung cancer need more investigation.

In this study, first we are interested in the expression of FGF16 in clinical lung cancer samples. We observed FGF16 overexpression in all 30 cases of human lung cancer tissues. Through online informatics software, we found that FGF16 might be one of the target genes of miR-520b. Interestingly, we observed a negative correlation of FGF16 with miR-520b in human lung cancer tissues. Meanwhile, we confirmed the inhibitory effect of miR-520b on the proliferation of lung cancer cells. Next, we are wondering whether miR-520b is able to affect the expression of FGF16 in lung cancer cells. Actually, we found that miR-520b could decrease the mRNA level and protein level in both A549 and H1299 lung cancer cells. In further investigation, we want to know how FGF16 is regulated by miR-520b in lung cancer. According to the prediction information of TargetScan, we cloned the seed region of 3′UTR of FGF16 mRNA binding to miR-520b into luciferase reporter vector. Our data showed that miR-520b could directly bind to 3′UTR of FGF16 mRNA to make its cleavage, leading to the reduction of FGF16 mRNA in lung cancer cells. Finally, we observed the inhibitory effect of miR-520b on lung cancer cell proliferation. Interestingly, we found that anti-miR-520b-induced augmentation in cell proliferation was obviously destroyed by RNA interference targeting FGF16 mRNA. It indicates that high FGF16 in lung cancer can serve as a biomarker and its regulating miRNA miR-520b may be used in clinical treatment for lung cancer.

In conclusion, FGF16 is highly expressed in lung cancer. miR-520b can directly interact with 3′UTR of FGF16 mRNA to induce its mRNA cleavage, resulting in its low expression. As a tumor suppressor, miR-520b can suppress the growth of lung cancer and its target gene FGF16 is able to reverse the inhibitory effect of miR-520b on lung cancer growth. Our finding can identify FGF16 as a novel biomarker for lung cancer and also provide the potential utility of miR-520-targeting FGF16 in lung cancer therapy.

## Appendix

**Table S1:** Clinical characteristics of lung cancer patient samples

No	Sex	Age	Pathology diagnosis
1	M	47	Lung cancer
2	M	52	Lung cancer
3	M	50	Lung cancer
4	M	63	Lung cancer
5	M	48	Lung cancer
6	F	69	Lung cancer
7	F	54	Lung cancer
8	F	49	Lung cancer
9	M	63	Lung cancer
10	M	68	Lung cancer
11	M	57	Lung cancer
12	F	41	Lung cancer
13	F	70	Lung cancer
14	F	56	Lung cancer
15	F	60	Lung cancer
16	M	52	Lung cancer
17	M	64	Lung cancer
18	M	61	Lung cancer
19	M	42	Lung cancer
20	M	67	Lung cancer
21	F	52	Lung cancer
22	M	58	Lung cancer
23	M	65	Lung cancer
24	F	59	Lung cancer
25	F	43	Lung cancer
26	F	66	Lung cancer
27	M	60	Lung cancer
28	F	51	Lung cancer
29	M	57	Lung cancer
30	M	62	Lung cancer

**Table S2:** List of primers used in this paper

Primer	Forward primer (5′–3′)	Reverse primer (5′–3′)
**Primers for RT-PCR and qRT-PCR**		
FGF16	CACCTTGAGATCTTCCCCAAC	TTCCTAGGTACAGGCCAGAG
GAPDH	CATCACCATCTTCCAGGAGCG	TGACCTTGCCCACAGCCTTG
MiR-520b	AAAGTGCTTCCTTTTAGAGGG	GCGAGCACAGAATTAATACGAC
U6	AGAGCCTGTGGTGTCCG	CATCTTCAAAGCACTTCCCT
**siRNA sequences**		
si-FGF16	CACCAGAAATTCACTCACTTT	
siCtrl	UUCUCCGAACGUGUCACGU	
